# Supplementary figures and images for: Data augmentation using image translation for underwater sonar image segmentation
Source: PLoS One. 2022 Aug 12;17(8):e0272602. doi: 10.1371/journal.pone.0272602 (PMC9374219; doi:10.1371/journal.pone.0272602)

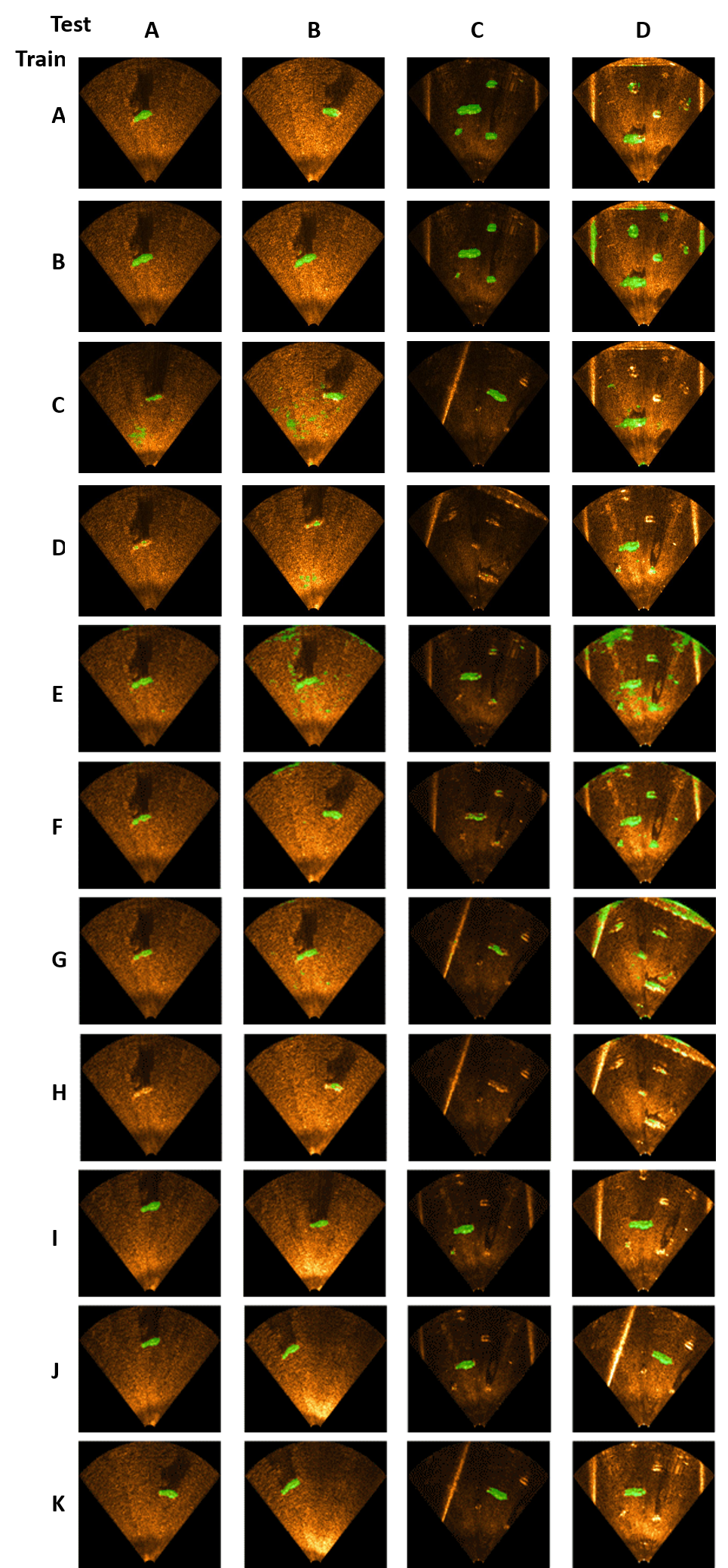

Supplement: S1 Fig — (A) Reservoir low sensitivity_Real, (B) Reservoir high sensitivity_Real, (C) Pool low sensitivity_Real, (D) Pool high sensitivity_Real, (E) Reservoir low sensitivity_Synth, (F) Reservoir high sensitivity_Synth, (G) Pool low sensitivity_Synth, (H) Pool high sensitivity_Synth, (I) T_Real, (J) T_Synth, (K) T_Aug. (TIF) [file pone.0272602.s001.tif]
