# Supplementary material for: Data augmentation using image translation for underwater sonar image segmentation
Source: PLoS One. 2022 Aug 12;17(8):e0272602. doi: 10.1371/journal.pone.0272602 (PMC9374219; doi:10.1371/journal.pone.0272602)
Supplement: Attachment — Submitted filename: PLOS ONE.docx [file pone.0272602.s007.docx]

In this manuscript, the authors used the Pix2Pix Generative Adversarial Network for image translation and then leverage it for data augmentation. Then a Fully Convolutional Network is used for image segmentation. The main aim of this research is to avoid over-fitting the model. Due to the extensive research conducted recently, it is absolutely necessary to pay attention to the following.

1- I suggest you have a brief overview of the related work on data augmentation and image segmentation. You can transfer a subsection of the "Introduction" to the "Related work" section.

2- The whole manuscript is full of abbreviations without defining them. Please define them before use.

3- Please, describe the details of the neural network architecture used. Includes the number of layers, neurons and activation functions and so on.

4- Please, describe in detail the data used. Includes the number of real data, synthetic data, dimensions of images and so on.

5- Please describe how to combine synthetic and real data to make augmented data.

6- What is the advantage of using FCN? That convinces us to augment data to learn the model.

7- Please provide a comparison without the use of incremental data to show that your method prevents over-fitting
