# Supplementary material for: Data augmentation using image translation for underwater sonar image segmentation
Source: PLoS One. 2022 Aug 12;17(8):e0272602. doi: 10.1371/journal.pone.0272602 (PMC9374219; doi:10.1371/journal.pone.0272602)
Supplement: Attachment — Submitted filename: REBUTTAL LETTER_PLOS ONE_To_Reviewer2.docx [file pone.0272602.s008.docx]

REBUTTAL LETTER

- Manuscript Number: PONE-D-22-11679R1
- Title: Data Augmentation Using Image Translation for Underwater Sonar Image Segmentation
- PLOS ONE

Dear Reviewer 2,

We would like to thank you and the reviewers for thorough reading of our manuscript and the comments which helped us to enhance the quality of the work. Their constructive comments are well-received and highly appreciated. We have carefully considered the reviewers' recommendation to further improve the clarity and quality of our manuscript. We hope that the revision made have improved the manuscript at all levels, and that the changes made in an attempt to address the comments of the reviewers are satisfactory.

Comments from Reviewer 2

| **Overall Comments** | |
| --- | --- |
| This paper has been to improve Underwater Sonar Image Segmentation by data augmentation and reduce the limitations of previous methods. However, before publishing the paper, I suggest some minor revisions, as follows: | |
| 1 | **Comment 1:**  In the abstract, please mention the exact amount of improvement and the metric that has been improved. |
|  | **Response:** Thank you for pointing this out. We agree with this comment. We will add content about the exact amount of improvement and the metric that has been improved in abstract. Before \| **Abstract** In underwater environment, the study of object recognition is an important basis for implementing an underwater unmanned vessel. For this purpose, abundant experimental data to train deep learning model is required. However, it is very difficult to obtain these data because the underwater experiment itself is very limited in terms of preparation time and resources. In this study, the image transformation model, Pix2Pix is utilized to generate data similar to experimental one obtained by our ROV named SPARUS between the pool and reservoir. These generated data are applied to train the other deep learning model, FCN for a pixel segmentation of images. The original sonar image and its mask image have to be prepared for all training data to train the image segmentation model and it takes a lot of effort to do it what if all training data are supposed to be real sonar images. Fortunately, this burden can be released here, for the pairs of mask image and synthesized sonar image are already consisted in the image transformation step. The validity of the proposed procedures is verified from the performance of the image segmentation result.; After \| **Abstract** In underwater environment, the study of object recognition is an important basis for implementing an underwater unmanned vessel. For this purpose, abundant experimental data to train deep learning model is required. However, it is very difficult to obtain these data because the underwater experiment itself is very limited in terms of preparation time and resources. In this study, the image transformation model, Pix2Pix is utilized to generate data similar to experimental one obtained by our ROV named SPARUS between the pool and reservoir. These generated data are applied to train the other deep learning model, FCN for a pixel segmentation of images. The original sonar image and its mask image have to be prepared for all training data to train the image segmentation model and it takes a lot of effort to do it what if all training data are supposed to be real sonar images. Fortunately, this burden can be released here, for the pairs of mask image and synthesized sonar image are already consisted in the image transformation step. The validity of the proposed procedures is verified from the performance of the image segmentation result. **In this study, when only real sonar images are used for training, the mean accuracy is 0.7525 and the mean IoU is 0.7275. When the both synthetic and real data is used for training, the mean accuracy is 0.81 and the mean IoU is 0.7225. Comparing the results, the performance of mean accuracy increase to 6 %, performance of the mean IoU is similar value.**; Location \| 1 page, “Abstract”. |
| 2 | **Comment 2:**  Organize the article in a standard format. Some sections, including sections 3 and 4, can be merged into one section. |
|  | **Response:** We agree with this comment. So, we will make merge some sections into one section.- Before \| **-**; After \| **Environmental Conditions of Underwater Sonar Image Dataset** We constructed datasets in two actual underwater environments using TELEDYNE BlueView M900-90 sonar to train the image translation NN and the semantic segmentation NN. The sonar had a frequency of 900 kHz, a beam width of 90° in the horizontal direction and 20° in the vertical direction, and a detection range of 100 m. The first underwater environment was a reservoir Fig. 6a. In this environment, we sunk a mannequin to the bottom of the reservoir, and then attached the sonar to the boat to obtain the data. The second environment was a water pool Fig. 6b. The pool had a width of 10 m, a length of 12 m, and a depth of 1.5 m. After sinking a mannequin with other artificial objects, such as a box and tire, to the bottom of the pool, we attached the sonar to SPARUS [23], an unmanned underwater vehicle, to obtain the data. When acquiring data in each environment, we adjusted the sensitivity of the sonar to create two conditions in each environment (reservoir high sensitivity - RHS, reservoir low sensitivity - RLS, pool high sensitivity - PHS, and pool low sensitivity - PLS) as shown in Fig. 7. NVIDIA T4 GPU and Keras package were used to train the image translation NN and semantic segmentation NN. We trained four image translation NNs to generate the synthetic USI for the datasets of the four conditions. In the training dataset for each environment, a binary mask and a USI are paired up, as shown in Fig. 4, and there are 200 paired images with a size of 512 × 256. So, real USI used is a total of 800 pages. The synthetic USI generated is 200 pages for each type, for a total of 800 pages with a size of 640 × 480. The image translation NN was trained using the training dataset with Adam optimizer for 150 epochs, and the hyperparameters of the sub-NNs, G and D, were as shown in Table 1. The semantic segmentation NN was trained to classify the pixels occupied by the object from the background in the USI based on the real datasets of the four conditions and the synthetic datasets of four conditions generated through the image translation NN. The training dataset for each condition consisted of a set of paired binary masks and USI as shown in Fig. 4. There were 200 paired images, each having a size of 640 × 480. The following table shows the description of the eight combined datasets used to train the semantic segmentation NN. The semantic segmentation NN was trained using the eight training datasets with Adam optimizer for 200 epochs, and the hyperparameters were shown in Table 2. Based on the eight datasets Table 3, we constructed three training datasets Table 4 to validate the effectiveness of the data augmentation using the synthetic USI, which were created using the image translation NN. The T Real dataset is a combination of the real datasets of the four conditions, and T Synth is a combination of the synthetic datasets of the four conditions. On the other hand, T Aug is a combination of the real datasets and synthetic datasets. When the semantic segmentation NN was trained using these datasets, the same number of paired images was used through uniform sampling. When the semantic segmentation NN was trained using the three training datasets, the hyperparameters used were the same as those used when the eight training datasets shown in Fig. 2 were used.; Location \| 5 page line 153-195. |
| 3 | **Comment 3:**  It is necessary to provide an architecture of the proposed method in your work. |
|  | **Response:** Thank you for this suggestion. We agree with this comment and describe the details of the neural network architecture used. – Before \| **-**; After \| “*G* consists of 8 encoder blocks and 8 decoder blocks each. In each block of the encoder, a convolution layer is used, the method of normalization is a batch norm, and activation function is Leaky ReLU. In each block of the decoder, transposed convolution layer and dropout is used, a method of normalization is the batch norm, and activation function is ReLU. *D* consists of 3 blocks. Each block uses a convolution layer, batch norm as a method of normalization, and Leaky ReLU as an activation function.” “A fully convolutional networks (FCN) is applied for the USI segmentation. A FCN is built by modifying VGG16 [22], a well-known NN used in image classification. The fully connected layers in VGG16 [22] are removed, and then 1×1 convolution layers, upsampling layers, and skip-connections [19] are added to facilitate dense prediction that outputs the pixel-level classification result with the same size as the input image”; Location \| 4 page, 111-116 line, 4 page, 123-127 line. |
| 4 | **Comment 4:**  In the result section, various tables have been provided. However, it is not discussed comprehensively. |
|  | **Response:** Thanks for this suggestion. We agree with this opinion. We will add discussion of experimental results in Conclusions and Future Works. Before \| **-**; After \| “If the proposed data augmentation method using the UIT is used, a large amount of synthetic USIs that are similar to real USIs can be created to train the semantic segmentation NN, even if the size of the real USI data is small. S1 Fig is the qualitative semantic segmentation result of Table 5 and 6. S1 and S2 Tables are quantitative semantic segmentation results that extend Tables 5 and 6. S1 Fig shows the results of training the semantic segmentation NN using the single types of training datasets separately. If the data used for training and testing have the same ’Environment’ and ’Sensitivity’ among the data types, the performance of Semantic Segmentation is good. On the other hand, when the types of data were different, the results of semantic segmentation was that the object of interest could not be recognized or the part that was not the object of interest was recognized as an object. These results are obtained when training with single type data, and when augmented data as shown in Table 4 is used for training, image segmentation performance is improved as shown in S1 Fig. Additionally, if the semantic segmentation NN is trained using both the real USIs and synthetic USIs, the performance of semantic segmentation can be improved compared with when it is trained using only the real USIs. Furthermore, as shown in S1 Fig, S1 and S2 Table, semantic segment results are good even when using only synthetic USIs when training models, showing the possibility that only synthetic USIs can be used as training data”; Location \| 9 page, 251-270 line. |

 Again, thank you for giving us the opportunity to strengthen our manuscript with your valuable comments and queries. We have worked hard to incorporate your feedback and hope that these revisions persuade you to accept our submission.

Sincerely,

Sejin Lee, Associate Professor

Div. of Mechanical and Automotive Engineering, Kongju National University

1223-24, Cheonan-daero, Seobuk-gu, Cheonan-si, 31080, Republic of Korea

Phone: +82-41-521-9259 Mobile: +82-10-4713-2692

Email: sejiny3@kongju.ac.kr
